# Factors Associated With Delayed Healing in a Study of the PrePex Device for Adult Male Circumcision in Kenya

**DOI:** 10.1097/QAI.0000000000000736

**Published:** 2016-05-24

**Authors:** Paul J. Feldblum, Elijah Odoyo-June, Robert C. Bailey, Jaim Jou Lai, Debra Weiner, Stephanie Combes, Catherine Hart, Shelly Fischer, Walter Obiero, Peter Cherutich

**Affiliations:** * Global Health Department, FHI 360, Durham, NC;; † Nyanza Reproductive Health Society, Kisumu, Kenya;; ‡ University of Nairobi, Nairobi, Kenya;; § University of Illinois at Chicago, Chicago, IL; and; ‖ National AIDS & STI Control Programme, Nairobi, Kenya.

**Keywords:** PrePex, Kenya, male circumcision, circumcision device, HIV prevention, wound healing

## Abstract

**Objectives:**

To explore factors associated with healing requiring more than 6 weeks after placement of the PrePex device for adult medical male circumcision.

**Methods:**

We enrolled 427 men ages 18–49 years in an observational study of PrePex at 1 urban and 2 peripheral clinics in western Kenya. Participants were scheduled for device removal at day 7 and a follow-up visit at day 42 (allowable range, 40–44) at which the provider recorded wound status, with complete healing defined as a dry wound without any scab, later confirmed by site investigator review of digital penile photographs. We performed univariate and multivariate logistic regression to explore associations between selected demographic, surgical, and follow-up factors and delayed healing (not healed by day 42 visit).

**Results:**

Of the 427 men, 341 completing a day 42 visit with physical examination and recorded healing status were included. Fifty-four percent of included men were healed by day 42 visit. Factors associated with delayed healing in univariate analysis and remaining significant in the multivariate analysis were as follows: age 25 years or older [odds ratio (OR): 1.8; 95% confidence interval (CI): 1.4 to 2.4], an adverse event by day 44 (OR: 1.4; 95% CI: 1.03 to 2.0), and severe pain during device removal (protective association: OR: 0.7; 95% CI: 0.5 to 0.99).

**Conclusions:**

Older age (25+ years), occurrence of an adverse event, and lesser self-reported pain at device removal were associated with delayed wound healing. If confirmed by larger surveillance studies, these results should be incorporated into the counseling given to male circumcision clients.

## INTRODUCTION

Voluntary medical male circumcision (VMMC) has been shown to reduce the incidence of HIV infection in men by about 60%, with the effect sustained for years.^1–3^ Most VMMC procedures are surgical, but the advent of devices for adult VMMC holds promise that programs integrating them can accelerate scale-up.^4^ The first device for adult VMMC to receive WHO prequalification status is the elastic collar compression device named the PrePex Male Circumcision System (hereafter PrePex).^5^ Clinical studies of PrePex have been conducted in Rwanda,^6–8^ Uganda,^9^ Kenya,^10^ and other countries.

We conducted an implementation pilot study of the safety of the PrePex device in routine service delivery in Western Kenya in 2013.^10^ One objective of the study was to determine the time to complete healing after PrePex placement. Wound healing after conventional surgical VMMC is by primary intention, meaning suturing is performed for wound closure, and the great majority of surgically circumcised men are completely healed within 6 weeks,^11^ so that WHO recommends 42 days of postprocedure abstinence.^12^ Healing following PrePex procedures is by secondary intention, meaning the wound is allowed to close naturally and tends to take longer than with surgical procedures.^13^ Longer healing time may mean that more men will resume sexual activity before complete healing and incur increased risks of HIV or sexually transmitted infection acquisition,^11^ although condom use at the time of resumption of sexual intercourse can mitigate those risks.^14^ Furthermore, uptake of VMMC could be jeopardized if a lengthier abstinence period is required.

Frequencies of healing by weeks after circumcision, and the mean/median days to healing, are often presented in clinical VMMC reports. Less commonly analyzed are factors associated with healing time. Knowledge of predictors of slower healing would be useful to inform policy regarding postplacement abstinence and to tailor counseling messages in VMMC programs. We identified Kenya PrePex study participants who were or were not completely healed at 6 weeks and examined factors associated with delayed healing in the cohort.

## METHODS

The implementation study is described in detail elsewhere.^10^ Briefly, our prospective PrePex study was conducted at 3 clinics in Nyanza Province, Kenya. Inclusion criteria were as follows: ages 18–49 years, HIV-uninfected, in good general health, clinically free of sexually transmitted infection, willing to provide contact information and written informed consent. A man was excluded from participation in the study if his penis did not fit any of the 5 PrePex sizes or he had a medical contraindication to VMMC or study participation. The primary objective of the implementation study was to assess the safety of PrePex procedures, and participants were evaluated for adverse events (AEs) at every visit. We also determined the time to complete healing after PrePex placement. Complete healing was defined as a dry wound without any scab. PrePex placements (day 0) and removals (scheduled at day 7) were performed as per the manufacturer's recommendations. The first 50 men underwent intensive follow-up with 6 study visits (at days 7, 9, 14, 28, 35, and 42 after device placement) to provide a detailed assessment of safety and healing. The remaining 377 men were scheduled for 2 follow-up visits, the first at day 7 after PrePex placement for device removal and the second at day 42 after device placement (ie, 35 days after removal) for wound inspection. We made multiple phone contacts with men who missed their day 42 visits to encourage them to attend. We followed men not completely healed by day 42 until complete healing.

### Outcome Variable

With the exceptions noted below, we limited the analysis to those participants who completed a day 42 visit and underwent a physical examination and healing assessment. Although the target date for the healing assessment visit was always 42 days after placement, not all men attended on their target date. We set the allowable visit window as days 40–44 and refer to a visit in that range as a day 42 visit. At that and every follow-up visit, each participant was certified by the provider as completely or not completely healed. The site investigator (E.O.-J.) reviewed digital penile photographs to confirm healing status recorded by the examiner. We defined healing as delayed if the wound was not completely healed at day 42 visit.

Two men were certified healed at day 36 and also completed visits at day 42, and we included them in the group of men healed by day 42. Seven men missed their day 42 visit but were examined at a later visit at which they were deemed not healed and thus were included in the day 42 delayed healing group.

### Independent Variables

We ascertained a series of potentially relevant baseline factors at the circumcision visit, including age, number of sexual partners in the last 6 months, current condom use (never vs at least some), and hypertension (≥140 mm systolic and/or ≥90 mm diastolic). Placement factors included clinic site (urban or peripheral) and the duration in minutes of the placement procedure. Follow-up factors included number of days postplacement when removal was performed, any detachment of foreskin from the penis observed before device removal, self-reported pain at removal on a scale of 0 (none) to 10 (worst possible),^15^ occurrence of an AE, making an unscheduled visit, and self-reported resumption of sexual activity before day 42 postcircumcision.

### Statistical Considerations

We reviewed the correlations among the selected factors to uncover potential collinearity that might render the eventual regression model unintelligible. After tabulating the distribution of the selected factors, we performed univariate logistic regressions to calculate the associations [odds ratios (ORs) and 95% confidence intervals (CIs)] between each factor and day 42 healing status (SAS PROC LOGISTIC, Cary, NC). Variables with a univariate *P* value <0.25 were considered for inclusion in a multivariate logistic regression model. Those factors with *P* < 0.10 were retained in the model as main effects. Factors whose removal from the model changed an OR estimate for a main effect by 15% or more were retained as confounders.^16^ Other than confounders, only those factors with nominal *P* value ≤0.05 were included in the final model.

We ran the final model again in a sensitivity analysis that included all 427 men in the implementation study cohort, with the assumption that participants previously excluded because of unknown healing status were not completely healed at day 42 visit, that is, a worst-case scenario.

## RESULTS

### Features of Men Included In and Excluded From the Analysis

Four hundred twenty-seven men were circumcised using the PrePex device in the implementation pilot study.^10^ For this analysis, the sample comprised (1) 334 men (78.2%) who completed a visit during day 42 visit window and had a physical examination with healing status recorded and (2) 7 men who were confirmed as not completely healed at a visit after day 42 and therefore could not have been healed at day 42. The total analysis population was 341, excluding the 86 men (20.1%) with unknown healing status at day 42.

Men included in the analysis were slightly older than those excluded (30% age 25 years or older vs 23%, respectively), less likely to exceed a hypertension threshold (31% vs 42%), more likely to report at least some condom use at baseline (50% vs 40%), more likely to be circumcised at the urban clinic site (29% vs 14%), and less likely to have a moderate/severe AE (3.5% vs 10.5%). For most placement and follow-up factors, the 2 groups were similar (percentage with early removals, foreskin detachment at removal visit, pain during removal, resumption of sexual activity before day 42 visit, occurrence of mild AE, unscheduled visit within 3 weeks of placement).

### Time to Complete Healing

Two-thirds of participants in this analysis completed their day 42 healing assessment visit on their exact target date. Of the 341 men analyzed here (53.7%), 183 were completely healed at their day 42 visit.

### Associations With Healing Status

Features of men in the analysis population by healing status are shown in Table 1. Data on moderate/severe AEs were sparse, as 9 men with such an event in the implementation study cohort were not eligible for this analysis because they did not complete a day 42 visit. We therefore analyzed a broader end point, occurrence of any AE regardless of severity (mild, moderate, or severe). Also, completing an unscheduled study visit was highly correlated with occurrence of an AE, so we elected to drop unscheduled visits from the modeling.

**TABLE 1.**
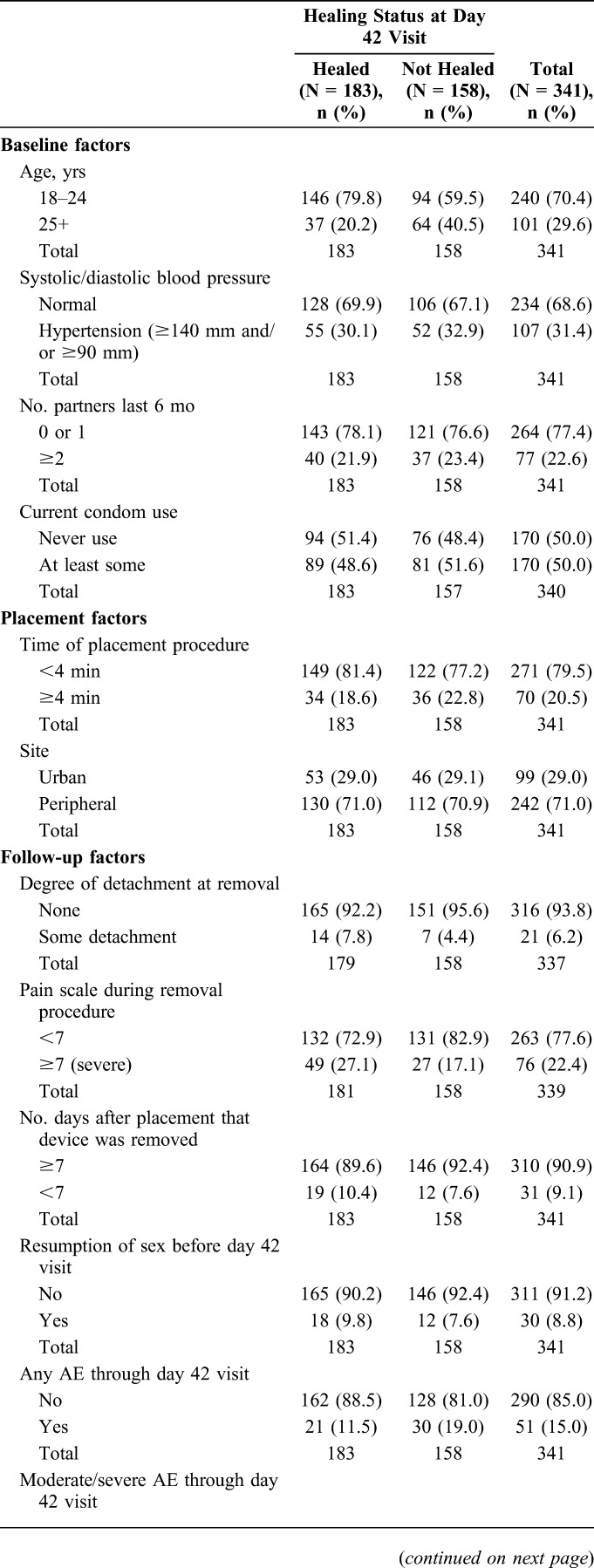

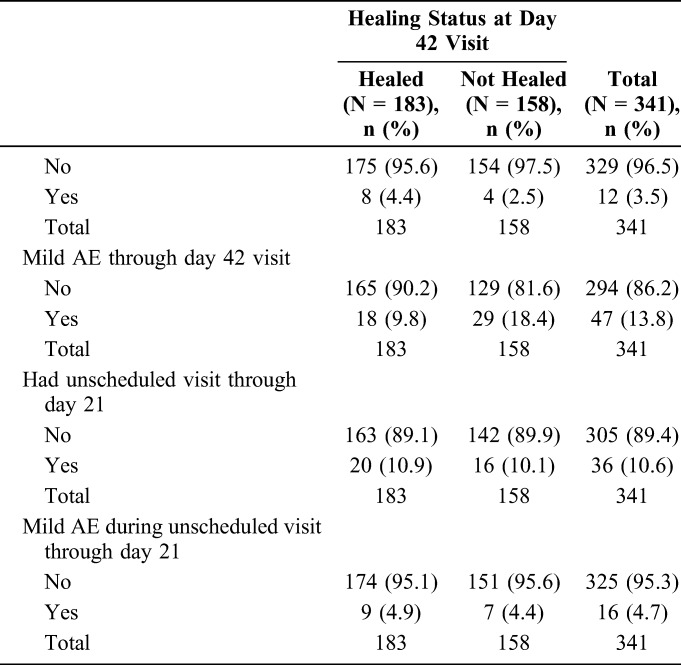
Selected Factors in the Analysis Population by Healing Status at Day 42 Visit

Four factors were identified for further analysis, with univariate *P* values <0.25: age 25 years or older vs age 18–24 years, any AE through day 42 visit, self-reported less intense pain during device removal, and any foreskin detachment noted at removal (Table 2). These 4 factors were entered into a multivariate logistic regression model.

**TABLE 2.**
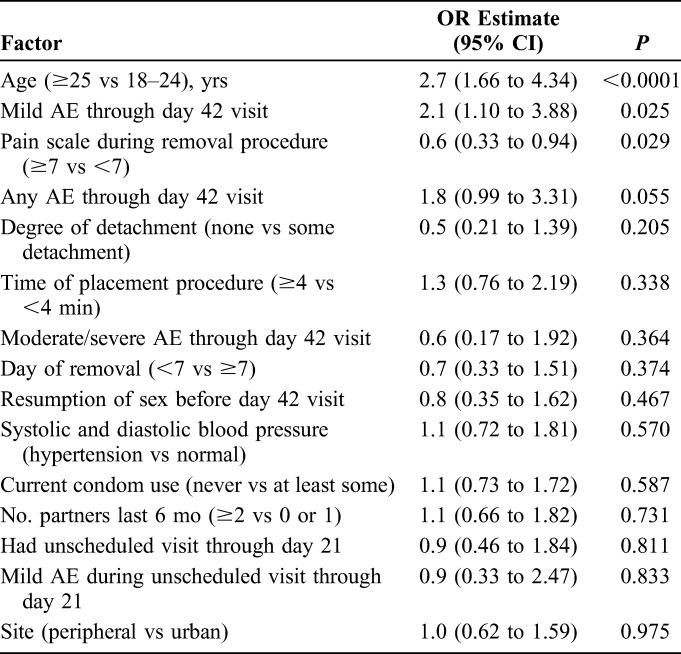
ORs, CIs, and *P* Values for Selected Factors and Delayed Healing in Univariate Logistic Regression

Three factors were retained in the final multivariate logistic model as main effects, with nominal *P* values ≤0.05 (Table 3): age ≥25 years (OR: 1.8; 95% CI: 1.4 to 2.4), AE of any severity by day 42 visit (OR: 1.4; 95% CI: 1.03 to 2.0), and pain score ≥7 during device removal having a protective association (OR: 0.7; 95% CI: 0.5 to 0.99). Urban vs peripheral clinic site was tested and retained as a confounder in the final model.

**TABLE 3.**
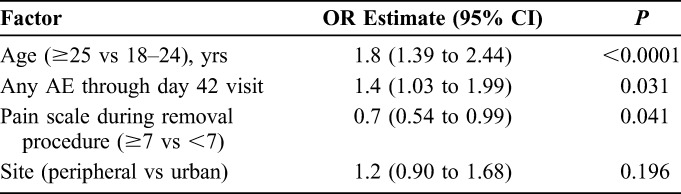
Factors Associated With Delayed Healing: Multiple Logistic Regression Model

### Sensitivity Analysis—Associations With Healing Status in Full Cohort

We conducted a sensitivity analysis on the full implementation study cohort of 427 men, assuming that men who exited without certified healing were healed later than day 42 visit. The results were similar to the main analysis and are not addressed further.

## DISCUSSION

This analysis aimed to elucidate some of the factors that lead to healing delayed beyond day 42 after PrePex circumcision. The strongest association was that older age (25 years or older) nearly doubled the odds of delayed healing compared with men aged 18–24 years. The effect of age has been observed in an even younger study population: a pilot study of PrePex use in Zimbabwe found that time to complete healing was shortest for participants aged 13–14 years, intermediate for those aged 15–17 years, and longest among adult men. Median time to complete healing in that study was about 1 week less for adolescents than for adults (K. Hatzold, MD, personal communication 2015). Also, Awori et al^17^ found that wound healing after ShangRing circumcision took longer among Kenyan adolescents than among younger children, although numbers were small in that pilot study.

Other age-specific healing data are scanty in the adult male circumcision literature. Rogers et al^11^ followed Kenyan men aged 18–35 years weekly for 7 weeks after forceps-guided surgical VMMC, and prompt tracing of study participants yielded a visit completion rate of 97.1%. They observed that 83.1% of men were completely healed by day 35 and 94.1% of men were completely healed by day 42. This compares with 50% healed by day 42 visit in the full PrePex Kenya implementation study cohort,^10^ and 54% healed in this analysis population. The factors reported by Rogers et al that were most associated with a reduced rate of healing were early postoperative infection and evidence of tight suturing, whereas older age had a modest nonsignificant impact on the healing rate. We observed no postplacement infections in the PrePex implementation study^10^ and in fact infection appears to be rare with this device.

We also found that having an AE occurring before day 42 was associated with delayed healing. Similar to the sparseness of published data on healing by age, little information is available regarding AEs and time to healing. One Kenyan study of surgical circumcision procedures found that adult men had a higher rate of moderate/severe AEs than adolescents.^18^ It is intuitive that wound disruption would be associated with slower healing, although prompt suturing of a dehiscence should accelerate initially slow healing.

The reason that lesser pain at device removal was associated with delayed healing is unclear. We did not record the brief but occasionally intense PrePex removal pain as an AE. Higher pain at removal was not correlated with pain AEs during subsequent follow-up; only 2 of the men reporting intense removal pain had a pain AE recorded. It is possible that men with a lower pain threshold who reported greater pain at removal differed in their wound care. There were differences in proportions of men reporting pain between the urban and peripheral clinics (controlled in our model) and differences in pain reported by men who were circumcised by clinical officers vs nurses. These differences might be related to variations in removal or other techniques, despite our efforts to standardize procedures.

A weakness of the implementation pilot study was that a large proportion of participants (24%) exited follow-up without information about their healing status. That loss of information was not relevant to this analysis because we restricted the analysis population to participants with a known healing status at day 42 visit window. Participants who were not completely healed at a physical examination performed after that point could safely be classified as slower to heal, even if they exited the study without complete healing. We also assessed the potential impact of those losses by conducting a sensitivity analysis in which all men who exited without certified healing were assumed to be healed later than day 42 visit, with no impact on the observed associations.

Another potential limitation of this analysis is the classification of healing status because healing assessments are somewhat subjective and can vary among male circumcision (MC) providers and between clinics.^19^ However, our criteria for complete wound healing (dry wound without any scab) were simple and unlikely to be misclassified. Moreover, the site investigator reviewed penile photographs, case report forms, and clinic notes to make final decisions on healing, several of which were overturned, thus adjudicating interobserver variation in those assessments.

Finally, our data collection lacked several items that appear to be relevant to the healing process. The implementation pilot study lacked in-depth information on resumption of sexual activity; one qualitative study found that one quarter of men resumed sex before 6 weeks after surgical circumcision,^20^ higher than the 9% we found in this analysis. Nor did we have information on marital status, which has been associated with resumption of sexual activity before complete wound healing.^14,21^ We did not inquire about behavioral factors including alcohol intake and physical activity level. We did not collect systematic data on the degree of circumcision experience of the providers in the study, a factor that is related to AE rates after surgical techniques.^22^ All of the providers used in this study had performed more than 1000 circumcisions using the forceps-guided method, but their experience with the PrePex device was limited. Finally, the PrePex implementation pilot study was restricted to HIV-uninfected men, so we could not compare healing and its predictors according to HIV status. Our analysis could not address the issue of whether delayed healing increases the risk of HIV acquisition or transmission, plausible as that appears.

This analysis and its source implementation study^10^ support the assertion that wound healing is delayed using the PrePex device compared with surgical circumcision. This is but one of multiple reports on the PrePex device and the similarly well-studied ShangRing device^19^ that have produced consensus that wound healing by secondary intention after device MC takes 1–2 weeks longer than healing after surgical MC.^13^ Healing by secondary intention has been shown to take longer than by primary intention for other surgical wounds.^23,24^

Longer healing time than after surgical MC will be an added challenge for programs that integrate devices for VMMC and counsel men to remain sexually abstinent until healing is complete. Some men may find circumcision with a device unacceptable for this reason; others who do have a device circumcision may resume sex before the longer healing period is complete. Practices that can reduce the extended healing period will be valuable for increasing the acceptability of devices and reducing the risks of post-MC disease transmission. Counseling on postplacement and postremoval care has evolved for the relatively novel PrePex device, with the addition of newly revised recommendations for penile hygiene during device wear.

This was a hypothesis-generating analysis, so that some results could represent random fluctuations. But, if our results are confirmed in analyses of large surveillance cohorts during PrePex scale-up, the factors we found to be associated with delayed healing, particularly older age and AE occurrence, should help to tailor the counseling of men who receive medical male circumcision using the PrePex device.
